# Closure and the Critical Epidemic Ending

**DOI:** 10.1484/J.CNT.5.128875

**Published:** 2022

**Authors:** Arthur Rose

**Affiliations:** Wellcome Centre for Cultures and Environments of Health, University of Exeter, Exeter, UK

**Keywords:** 20th C. History, Epidemics, Historiography of Science, Medical Humanities, Narrative, Philosophy of History

## Abstract

“An epidemic has a dramaturgic form,” wrote Charles Rosenberg in 1989, “Epidemics start at a moment in time, proceed on a stage limited in space and duration, following a plot line of increasing and revelatory tension, move to a crisis of individual and collective character, then drift towards closure.” Rosenberg’s dramaturgic description has become an important starting point for critical studies of epidemic endings (Vargha, 2016; Greene & Vargha, 2020; Charters & Heitman, 2021) that, rightly, criticize this structure for its neatness and its linearity. In this article, I want to nuance these criticisms by distinguishing between the term Rosenberg uses, “closure,” and its implicature, “ending.” I aim to show how many of the complications ensuing between the different forms of ending imagined may well be resolved by assessing whether they bring closure or not.

“An epidemic has a dramaturgic form,” wrote Charles Rosenberg in 1989, “Epidemics start at a moment in time, proceed on a stage limited in space and duration, following a plot line of increasing and revelatory tension, move to a crisis of individual and collective character, then drift towards closure.”^1^ Rosenberg’s dramaturgic model often appears as the starting point for historians reflecting critically on the endings of epidemics.^2^ These reflections criticize the model’s neatness and linearity in order to signal a disconnect between historical conditions that frustrate easy notions of ending—the ongoing imprints of the disease “on the bodies of survivors, societies and cultures” after an epidemic has been declared over—and a historiography that often relegates these imprints to “epilogues.”^3^ But their use of the model is largely pragmatic. Rosenberg’s dramaturgy, as it appears in these texts, serves less as a target of criticism than as an exemplar of a narrative arc.

Adapting Mary S. Morgan’s description of narrative, Rosenberg’s “dramaturgy” “create[s] a productive order amongst materials with the purpose to answer why and how questions.”^4^ It offers, in its “drift towards closure,” “an identification of an epidemic’s end as the point when the urgency of the disease outbreak has sufficiently diminished.”^5^ As such it becomes a foil to other possible endings, for different people at different times. “Not only does a given epidemic ‘end’ at different times in different locations, and for different groups in the same location,” Erica Charters and Kristin Heitman remind us, “but also for different academic disciplines: epidemiologists, anthropologists, policymakers, and historians follow different parameters to gauge the decline and end of epidemics.”^6^ By raising different possible endings, these historians foreground the people and organizations who decide on the end of an epidemic, as it occurs and after, and those excluded from this decision-making.

In the extended public engagement with epidemiological science during the COVID-19 pandemic, these interventions have particular political, social, and ethical urgency. They help to explain why different stakeholders might experience different feelings of resolution or irresolution. But resolution depends more on closure, the term Rosenberg uses, than on ending, which is favoured by his respondents. Interpretations of endings, made by policy-makers, populations, and public health experts in the moment, and by historians after the fact, must often balance a desire for closure against those facts that disrupt it. The aim of this essay is to bring the relation between closure and ending to the fore, and so offer a conceptual tool for historians to explain why some endings bring resolution and others do not. In what follows, I first clarify what I mean by ending and closure. Then I rehearse some of the arguments used by historians engaging with Rosenberg and critical epidemic endings, to show how their engagements with the writing of epidemic history might benefit from a more explicit distinction between ending and closure. Raising this distinction is not simply a problem of terminology. It has consequences for both historical research and the writing of historical narrative. Accordingly, I consider how narrativist and post-narrativist approaches to the philosophy of history have responded to the problem of closure.

## I

“An end,” writes Aristotle in the *Poetics,* “is that which itself naturally occurs, whether necessarily or usually, after a preceding event, but need not be followed by anything else.”^7^ It is the moment when things stop. Closure, as Tobias Klauk et al. have argued, describes a “feeling of finality,” an “impression that the story is complete,” leaving “no further questions” concerning its content.^8^ Rather than the moment when things stop, it describes a formal resolution, wherein one feels one’s questions have been answered and one’s expectations satisfied.

As a provisional definition, I want to suggest that endings in epidemic histories present interpretative acts of terminus. These acts are made by authorities, underwritten by collective agreement, and shaped by external conditions. But they also operate through the decisions of working historians, who, through heuristics, critique, and interpretation, establish contingent end points for historical periods. I emphasize “interpretation” and “decision” because, insofar as critical accounts of epidemic endings problematize these endings as given, they implicitly depend upon rival interpretations of what facts or testimonies are materially relevant, and upon the historical actors deciding on these matters.

Closure, however imbricated in this understanding of ending, remains distinct from it, since it describes the degree of resolution these endings bring. When the participants in the historical moment experience this resolution, we call it psychological closure, and when it occurs in a historical narrative, we call it narrative closure. In both cases, this resolution can be broken down into the fulfilment of expectations and the answering of questions.^9^ Does the narrative (or the circumstance) satisfy our expectations of what such an end might look like, and does it answer many, most, or all the outstanding questions that we might have? Insofar as an ending may satisfy our expectations without necessarily resolving all our questions (or vice versa), it may have an end without (complete) closure. Or, a condition may satisfy our expectations and answer our questions without ending. In such a case, we can say this is a closure without end.

This difference means there can be endings without closure and closure without endings. A disease might be eradicated, while leaving no firm feeling of finality for those who continue to bear its scars. Here, we might identify an ending without any ready experience of closure. Conversely, when a disease, once epidemic, begins to be accepted as endemic by policy-makers, populations, and public health experts, this acceptance gives the impression that the epidemic is complete without the disease ever really stopping its spread. In this example, closure happens without any discernible ending. But cases are rarely so clear cut.

Imagine, in the wake of a devastating epidemic, a successful eradication programme leaves the living to bury their dead, uncertain of what happened and dissatisfied with whatever actions were taken, but, on the whole, relieved the epidemic is “over.”^10^ Although the epidemic has ended, the closure each actor experiences differs. The epidemiologist or public health official announces the success of the programme, an act that becomes synonymous with the end of the epidemic. Supported by research findings and avowals of professional judgement, this announcement presents an explanatory resolution of the crisis that produces narrative closure. But the officials may have some doubts about the programme’s efficacy. This lingering uneasiness translates into a lack of psychological closure for the officials, if not for the public that believes in them. If such doubts are articulated, and these articulations become public knowledge, this lack of psychological closure may call into question the story’s narrative closure. Likewise, those people left scarred by the program’s effects may not feel their expectations satisfied or questions answered, even if they accept that support for the crisis has ended. Conversely, they may be all too ready to announce the end, perhaps even before the information concurs with such an assessment. Here, psychological closure can advance declarations of an end, outflanking the best intentions of those epidemiologists presumed to know.

The historian, coming after, might draw on the epidemiologist’s notes to concur with their judgement, only to find a disparity between their public support for an ending premised on narrative closure and a private record of doubt and scepticism more closely aligned with those who feel left behind. In writing about epidemics, historians may agree with the public declarations, especially when made by organizations like the WHO, or epidemiologists or political leaders: in such cases, the declared ending maps closely to the narrative closure of the historical text. They can problematize these public declarations by revealing their authors’ private reservations, expressed in diaries or letters: although the story of the event retains its narrative closure, the historical text distances itself from that story by recalling a relative lack of psychological closure for actors at the time. Or, as in the case of the critical reflections of epidemics’ ending, the relative artificiality of such declarations presents a target for historical revisionism, whereby new questions may be raised and expectations frustrated: the linear narrative defining an epidemic’s rise and fall, once treated as closed, is wrenched open once more.

## II

Although Rosenberg uses the term “closure,” critical accounts of his essay have tended to interpret this as “ending,” which they go on to complicate in productive ways. Writing in 2016, Dora Vargha summarizes the defining questions for the study of epidemic endings, as “when and for whom diseases end, what happens when the end fails to come, who gets to determine the end and who gets left behind, how a focus on endings shape health policies and how we can critically rethink the temporalities of epidemics.”^11^ In the context of COVID-19, Jeremy A. Greene and Vargha have observed that epidemics “have two faces.” These faces, “the biological and the social, are closely intertwined, but they are not the same. The biological epidemic can shut down daily life by sickening and killing people, but the social epidemic also shuts down daily life by overturning basic premises of sociality, economics, governance, discourse, interaction.”^12^ Inspired by efforts to find some organizing structure for the pandemic in Rosenberg’s dramaturgy, an entire special issue of *Bulletin of History of Medicine* re-examined his essay in light of the COVID-19 pandemic.^13^ And, while scholars dissected its blindness to conditions outside its North American context and its overly schematic reliance on a culturally specific dramaturgical sequence, these criticisms were offered, for the most part, as supplementary efforts to expand the model, rather than jettisoning it entirely.^14^ Charters and Heitman also outlined two possible endings for epidemics: biological eradication and a cultural acceptance that effectively normalizes conditions as “endemic.”^15^ As Greene and Vargha put it, endings that occur through cultural consensus tend to be asymptotic, where the graphic representation of actual cases is concerned: “rarely disappearing, but rather fading to the point where signal is lost in the noise of the new normal—and even allowed to be forgotten.”^16^

When it comes to endings, these different approaches tend to challenge Rosenberg’s model by offering exemplary cases that complicate its neat linearity. These examples pull together a combination of physical constraints (from the factual circumstances of virology and epidemiology to the effects of geography and systemic social inequalities) and collective consensus (the technocratic work of the WHO, the “collective amnesia” of the general public, and the social engineering of political, economic, and cultural decision-makers). As I have given them, the categories blur into each other, but this concerns me less than what is not given: a purely formal definition on what an ending is. When Vargha and Greene aim to “reconsider what we mean by the talk of ‘ending’ epidemics,” and subsequently propose to “take a step back and reflect in detail on what we mean by ending in the first place,” it is telling that this meaning is made through the “many forms” that “the history of epidemic endings has taken.”^17^ The endings, subsequently enumerated, are based on concrete historical examples.

Although such “thinking through cases” is, of course, useful, it leaves unstated what is meant by ending as such.^18^ In using Rosenberg, the essays each assume a conventional understanding of ending that they go on to challenge. When the writers examine epidemic endings, they leave ending as a concept largely untouched. Or, to put it another way, by relying on a typology of endings to demonstrate how epidemics are framed “within cycles of disease and with a multiplicity of endings,” these accounts of epidemic endings do not really address the notion of ending that they seek to displace.^19^ This is why I believe a formal distinction between ending and closure helps to elucidate what remains implied in the case literature.

The only substantive reference to closure, across this emerging literature, appears in Guillaume Lachenal and Gaetan Thomas’s examination of dramaturgic form, wherein the “endlessness” of actual epidemics is opposed to the apparent panacea of their closure: “Instead of events oriented toward their own closure, epidemics might be approached as unsettling, seemingly endless, periods during which life has to be recomposed.”^20^ This opposition exemplifies a common criticism of Rosenberg: he occludes variable endings and geo-cultural disparities. But it also highlights the equally common tendency to conflate closure and ending. This is a conflation because “ending” in these accounts does not simply describe definite external measures of terminus; it includes the feelings of satisfaction and completion that accompany these measures. When Lachenal and Thomas qualify the “endless” periods of epidemics as “seemingly,” they imply that it appears this way to a someone, whose dissatisfaction is conditioned by a period’s failure to fulfil expectations or answer questions. In this regard, endlessness is an impression that stems less from the lack of an ending than from a lack of closure.

Now, as H. Porter Abbott has argued, narrative is marked “almost everywhere by its *lack of closure.*” This process of deferring closure, also known as suspense, is often a desirable feature in narratives, as is its counterpart, the upsetting of expectations, or surprise. “All successful narratives of any length are chains of suspense and surprise that keep us in a fluctuating state of impatience, wonderment, and partial gratification.”^21^ Many, in their direct experiences of “historical time” during the COVID-19 pandemic, may find this lack of closure less pleasing than, say, the deferred closure that future histories of this period will undoubtedly use. In one possible interpretation, then, closure would appear to be far more relevant for the writing, or “representation,” of the epidemic, than for the heuristics, critique, and interpretation at work in the methodical analysis of its unfolding. In his classical philosophy of history, J. G. Droysen suggests just such a distinction between writing and method, the better to establish the historian’s analysis as separate, and separable, from their techniques of representation, or style.^22^ For this reason, we might conclude that Rosenberg’s critics are right when they implicitly suspend closure from their examination of actual historical events: the *material* differences lie in the way people construe endings, and so endings serve as a more important topic of inquiry.

What this conclusion ignores, however, is the extent to which criticisms of Rosenberg’s model rely on the shaping force of the narrative model itself, whether through comparisons between the dramatic arc and William Farr’s epidemic peak, the replacement of Rosenberg’s Grecian dramaturgy with one based on other dramatic forms, or the recognition that, in some places, epidemics are experienced as periodic cycles with no final terminus.^23^ “Narrative,” as Morgan puts it, “is how the relationships amongst their materials become known to them.”^24^ Indeed, Charters and Heitman insist that,

Focusing on how epidemics end is not simply a conceptual exercise, a narrative trick in which one views disease from a new perspective. Instead, it reveals the social and political processes by which a disease becomes endemic—that is, accepted—as well as who participates in that process and who is excluded.^25^

In making this claim, they imply that the narrative model plays a critical role in the interpretative act. But, by linking the endemic to “acceptance,” they are also implying, however obliquely, that these material processes set up expectations that can be satisfied or frustrated. So, even if we appear only to be talking about endings, these endings are still being mediated by different forms of closure.

This raises the question of whether my distinction between closure and ending really needs to be developed in such elaborate terms. Surely, my argument might be better conveyed in 280 characters (or less), and to greater effect. “Great work on endings, but shall we unpick the ways these different examples grant or withhold closure?” To explain why it does, we might begin by bringing this work into closer alignment with the narrativist and post-narrativist approaches to the philosophy of history that arose in the wake of Hayden White’s *Metahistory* (1973). A more explicit engagement with these insights may help to understand why the implicit references to closure, as a general orientation towards endings, create complex problems as they blur across the methodological/representational divide.

## III

In *The Content of the Form* (1987), Hayden White opines that “what is represented in a narrativization of a sequence of historical events” is “the seizure by consciousness of a past in such a way as to define the present as a fulfillment rather than as an effect.” This “reveal[s] everything in it as a prefiguration of a project to be realized in some future.”^26^ To reframe White’s larger project in simple, even reductivist, terms, narrative fails to respect the distinction, marked in the scientific method, between the representation of history, through writing or other forms of presentation, and its interpretation. Narrative intrudes on interpretation, orienting it towards “fulfilment” (or closure). By proposing forms of history-writing that resist endings, the historians discussed above often gesture suggestively towards avoiding narrative closure: offering multiple, alternative ways of construing the end, recalling those for whom no simple end can be viable, and challenging the dominant narratives, usually determined by persons or organizations in positions of power and privilege, that propose clear and definitive endings. At first glance, we might imagine that White would find this self-aware engagement with the constraints of narrative all to the good. As Maria Grever writes, in a review of Kalle Pihlainen’s *The Work of History* (2017): “White particularly warned that the realist closure tends to domesticate and normalize the presentation of past events. The absence of closure reveals a narrative’s constructive and ideological nature, but it also provides room for reflection and discussion.”^27^ And, as Pihlainen, whose work offers some of the fullest engagement on White and closure, wrote almost 20 years ago, “closure causes discomfort”: “aesthetic closure causes discomfort because it is so clearly an *imposition* on the epistemological … moral closure causes discomfort because it is an evaluation of the other, and therefore a refusal to fully recognize [their] subjectivity.”^28^ The aesthetic form that Pilhainen describes, wherein closure restricts the epistemological possibilities of history, bears little resemblance to Abbott’s more technical description of satisfying expectations and answering questions. Doubtless this comes from the disciplinary differences: Abbott is concerned with a description of fictional narrative, whereas White and his followers make normative claims about historical narrative. But it might also explain how a seemingly technical matter of style can carry significant ideological baggage.

The attention to alternative endings betrays a certain anxious antipathy, not merely to “closure” as a narrative term, but to an ideological completeness inferred in reading Rosenberg’s model. Focused as it is on the United States during the AIDS epidemic, Rosenberg’s essay defends the epidemic as a local event and relies on oppositions between the developing and developed world that have, as Mariola Espinosa demonstrates, “little value.”^29^ A more expansive understanding, Espinosa argues, would explain how such events frequently play out on a regional or even global stage: after all, she wonders, why group the US with Western Europe over its more immediate Latin American neighbours? In identifying problems with Rosenberg’s “mode of explanation” and his “plot structure,” Espinosa gestures to a philosophy of history that, as White writes in *Metahistory,* “would appear only in the mode of explanation actually used to account for ‘what happened’ in the historical field and in the plot structure used to transform *the story actually told* in the narrative into *a story of a particular kind.”*^30^ Philosophies of history are *implicit* in the modes of explanation practicing historians use, not least in the ways they end up reproducing existing genres of scholarship when transforming the actual sequence of events into a historical narrative. That the historians are quite capable of addressing this transformation themselves, and in sophisticated terms, signals how scholarship has changed since White’s attack on vulgar realism. But it also confirms Carolyn J. Dean’s suspicion that, insofar as his legacy can be measured in mainstream history, it has “recast White’s attention to the language of representation as a form of critical self-awareness.”^31^ In offering their supplemental accounts as if they were “the story actually told,” replacing Rosenberg’s “story of a particular kind,” the historians signal that there is still more to be done to reconcile the practice of history with its philosophy.^32^ Or, in Dean’s analysis, “an openness to the diversity of argument may obscure other forms of methodological and professional consensus.”^33^

Within the emerging literature on epidemic endings, one site of implicit consensus appears to be that *a* narrative model might be useful. But, even as the literature affirms the interpretative value of narrative, it continues to depend upon concrete case examples, developed through archival research, to overrule narrative’s perlocutionary power. As such, it conditions the model with appeals to historical reality. The implicit post-narrativist sensibility at work here is not the thesis-driven philosophy history proposed by Jouni-Matti Kuukkanen.^34^ Rather, it resembles the post-narrativist framework proposed by Harry Jansen, where narrative and representation play a crucial role in understanding and interpreting historical events, while relying on distinct appeals to research and experiences of time.^35^ By defining endings through case examples, rather than a contingent definition, the historians repeat Louis O. Mink’s narrativist rule that the historical thesis is non-detachable: “What exemplifies [aspects of the past referred to in the narrative] and what is exemplified [the historical thesis] mutually depends on each other. Therefore, there is no such thing as a general historical thesis which is exemplified in different narratives.”^36^ At the same time, in the efforts to displace Rosenberg’s model, where it is too linear, schematic, or reliant on a single arc of expansion and contraction, the appeal is not, or not only, to alternative narratives.^37^ It relies on colligations that extend the epidemic beyond the strictures of the model.

According to W. H. Walsh, when historians formulate “inner connections between certain historical events” into “a single process, a whole of which they are all parts and in which they belong together in a specially intimate way,” this process, and its attendant rhetorical claims, is called colligation.^38^ When colligation plays a role in narrative ordering, or “configuration,” it assembles things “together under a label,” as an exemplary case.^39^ In the end of epidemics literature, these colligations allow the process of the epidemic—its affiliate events—to extend beyond what seems to be a conventional ending. As Morgan has argued, colligations do not create explanatory narratives, in and of themselves; rather, historians juxtapose several such colligates, in their essays, to prompt questions and offer answers.^40^ Since the epidemic’s discontents continue to arise after its end, the historians argue, these subsequent events, uncovered by further research, must be colligated into its process.

But, as Jansen argues, this appeal to research relies on an argumentative infrastructure that unfurls in the preparation phase and acts as a “hidden persuader” during the writing phase. This argumentative infrastructure develops because of “the epistemic values of colligation (such as exemplification, coherence, comprehensiveness/scope, and originality)” and “the perception of a continuous or a discontinuous time.”^41^ What Jansen signals are subtle inclinations towards narrative and psychological closure during historical research that ultimately influence the writing of history. Regarding colligations, we might emphasise the rhetorical role played by encapsulated histories of previous pandemics in the emerging literature. Paragraph-length accounts of 1918 influenza, typhoid, smallpox, or polio produce all the values of a bite-sized colligation, while their enumeration emphasizes the multiplicity of endings at work. So, while the juxtaposition of case examples, in the form of colligations of other epidemics, keeps the possible epidemic endings open, the colligations themselves represent each of those epidemics as having achieved a certain degree of narrative closure. For this reason, the writing of history often depends on a research phase punctuated by prior moments of narrative closure, even when it tends towards openness. A parallel argument may be made for the psychological closure that attaches to perceptions of epidemic time as “discontinuous” from normal, “continuous” time, which “resumes” once the epidemic is over. Recalling how forms of narrative and psychological closure may be influencing the “sense of an ending” that attaches to an epidemic, not simply in its writing but across the research period, may further support the study of epidemic endings.

My aim in this essay has been to extend discussions about epidemic endings by recalling the distinction between closure and ending. This distinction is generative, insofar as it prompts us to consider how the act of interpreting an ending may leave questions unanswered and expectations unfulfilled. To the questions already posed, about who decides an end and who is excluded from that decision, it adds: does the ending provide psychological and/or narrative closure to the participants, be they survivors, public health or political officials, or organizations, and how should these forms of closure be relayed by historians coming after? Dealing with these questions raises further questions for the writing of history: whether, when the historian relays a lack of closure in their materials, they develop a style that mimics this lack or bounds its description in more readerly narratives. What I have hopefully made clear is that such questions are not simply applicable for the larger narrative ordering of the book, monograph, or article; it permeates down to the paragraph-length case, a site where explanatory closure may deny the very openness implicit in asking how epidemics end.

## Footnotes

1 Rosenberg (1989, p. 279). Reprinted in Rosenberg (1992); Rosenberg (2020).

2 See, for instance, Vargha (2016); Greene & Vargha (2020); Charters & Heitman (2021).

3 Vargha (2016). See also Charters & Heitman (2021, pp. 212–213).

4 Morgan (2017, p. 86).

5 Charters & Heitman (2021, p. 212).

6 Charters & Heitman (2021, pp. 211–212).

7 Aristotle (1999, p. 55).

8 Klauk, Köppe, & Onea (2016, pp. 38–39).

9 Abbott (2013, p. 58).

10 On the complications of declaring an epidemic “over,” see Charters & Heitman (2021, p. 214).

11 Vargha (2016).

12 Greene & Vargha (2020).

13 Fissell, Greene, Packard, & Schafer. (2020).

14 See, in particular, Espinosa (2020); Sivaramakrishnan (2020); and Peckham (2020), for the former; and Peckham (2020); Lachenal & Thomas (2020); Vargha (2020); and Callard (2020), for the latter.

15 Charters & Heitman (2021).

16 Greene & Vargha (2020).

17 Greene & Vargha (2020).

18 See Forrester (1996), on what happens when we try to extrapolate from a case study, or, in Forrester’s provocative formulation, “if P, then what?” See also the Special Issue of *History of the Human Sciences*, reappraising Forrester’s essay, Millard & Callard (2020), in particular the contributions by Flexer (2020) and Morgan (2020).

19 Charters & Heitman (2021, p. 210).

20 Lachenal & Thomas (2020, p. 672).

21 Abbott (2013, p. 57).

22 See Rüsen (2020, p. 97).

23 See, respectively, Lachenal & Thomas (2020, p. 677); Peckham (2020, p. 665); Sivaramakrishnan (2020, p. 641).

24 Morgan (2017, p. 87).

25 Charters & Heitman (2021, p. 218).

26 White (1987, p. 149).

27 Grever (2020, p. 495).

28 Pihlanein (2002, p. 189).

29 Espinosa (2020, p. 636).

30 White (1973, p. 276).

31 Dean (2019, p. 1348).

32 See, for instance, Charters & Heitman (2021, p. 211), who, rightly, interrogate researchers for having “paid little attention to how epidemics actually end.”

33 Dean (2019, p. 1349).

34 Kuukkanen (2015).

35 Jansen (2019, p. 72).

36 Mink, quoted in Jansen (2019, p. 74).

37 Although, as in Peckham (2020), these too play a role.

38 Walsh (1967, pp. 25, 24). On the longer history of colligation, including its coinage by William Whewell (1840), see Cristalli & Sánchez-Dorado (2021).

39 Morgan (2017, p. 96).

40 Morgan (2017, p. 96).

41 Jansen (2019, p. 88).

## References

[R1] Abbott HP (2013). The Cambridge introduction to narrative.

[R2] Aristotle, Halliwell S (1999). Poetics.

[R3] Callard F (2020). Epidemic time: Thinking from the sickbed. Bulletin of the History of Medicine.

[R4] Charters E, Heitman K (2021). How epidemics end. Centaurus.

[R5] Cristalli C, Sánchez-Dorado J (2021). Colligation in modelling practices: From Whewell's tides to the San Francisco Bay model. Studies in History and Philosophy of Science.

[R6] Dean CJ (2019). Metahistory: The historical imagination in nineteenth-century Europe, by Hayden White. The American Historical Review.

[R7] Espinosa M (2020). Revisiting “What is an epidemic?” in the time of COVID-19: Lessons from the history of Latin American public health. Bulletin of the History of Medicine.

[R8] Fissell ME, Greene JA, Packard RM, Schafer JA (2020). Reimagining epidemics. Bulletin of the History of Medicine.

[R9] Flexer M (2020). If p0, then 1: The impossibility of thinking out cases. History of the Human Sciences.

[R10] Forrester J (1996). If p, then what? Thinking in cases. History of the Human Sciences.

[R11] Greene JA, Vargha D (2020). How epidemics end. Boston Review.

[R12] Grever M (2020). History writing without closure. History and Theory.

[R13] Jansen H (2019). Research, narrative and representation: A postnarrative approach. Historyand Theory.

[R14] Klauk T, Köppe T, Onea E (2016). More on narrative closure. Journal of Literary Semantics.

[R15] Kuukkanen J-M (2015). Postnarrativist philosophy of historiography.

[R16] Lachenal G, Thomas G (2020). Epidemics have lost the plot. Bulletin of the History of Medicine.

[R17] Millard C, Callard F (2020). Thinking in, with, across, and beyond cases with John Forrester. History of the Human Sciences.

[R18] Morgan MS (2017). Narrative ordering and explanation. Studies in History and Philosophy of Science.

[R19] Morgan MS (2020). “If p? Then what?” Thinking within, with, and from cases. History of the Human Sciences.

[R20] Peckham R (2020). The crisis of crisis: Rethinking epidemics from Hong Kong. Bulletin of the History of Medicine.

[R21] Pihlanein K (2002). Of closure and convention: Surpassing representation through performance and the referential. Rethinking History.

[R22] Rosenberg C (1989). What is an epidemic? AIDS in historical perspective. Daedulus.

[R23] Rosenberg C (1992). Explaining epidemics and other studies in the history of medicine.

[R24] Rosenberg C (2020). What is an epidemic? AIDS in historical perspective. Bulletin of the History of Medicine.

[R25] Rüsen J (2020). A turning point in theory of history: The place of Hayden White in the history of metahistory. History and Theory.

[R26] Sivaramakrishnan K (2020). Looking sideways: Locating epidemics and erasures in South Asia. Bulletin of the History of Medicine.

[R27] Vargha D (2016). After the end of disease: Rethinking the epidemic narrative. Somatosphere.

[R28] Vargha D (2020). Reconsidering the dramaturgy. Bulletin of the History of Medicine.

[R29] Walsh WH (1967). Philosophy of history: An introduction.

[R30] White H (1973). Metahistory: The historical imagination in nineteenth-century Europe.

[R31] White H (1987). The content of the form: Narrative discourse and historical representation.

